# Evaluation of potentially inappropriate medication in older patients with cardiovascular diseases—STOPP/START-based study

**DOI:** 10.3389/fpubh.2022.1023171

**Published:** 2022-12-22

**Authors:** Tzvetan Krustev, Petya Milushewa, Konstantin Tachkov, Konstantin Mitov, Guenka Petrova

**Affiliations:** Department of Organization and Economics of Pharmacy, Faculty of Pharmacy, Medical University of Sofia, Sofia, Bulgaria

**Keywords:** elderly patients, inappropriate prescribing, cardiovascular diseases, STOPP/START criteria, polypharmacy, older patients

## Abstract

**Objective:**

This study aims to evaluate the use of STOPP/START criteria in the identification of Potentially inappropriate medication and potential prescribing omissions in older patients with cardiovascular diseases in Bulgaria. Excessive morbidity and mortality has been linked to drug-related problems and increased use of healthcare services and is an understudied problem for Bulgaria.

**Materials and methods:**

A prospective, questionnaire-based study was conducted among 543 older patients across 25 pharmacies in Bulgaria. Socio-demographic characteristic, disease profile, symptoms, and medication data were collected. The questionnaire was developed for the purposes of the EUROAGEISM project. Out of all 543 patients, only those with documented cardio-vascular diseases were extracted and the medication profile per patient was evaluated for Potentially inappropriate medication (PIMs) and potentially prescribing omissions (PPOs) using STOPP/START criteria version 2. In addition, several risks for potentially inappropriate prescribing (PIPs), PPOs and PIMs were calculated with the focus being on the Odds and Risks to develop a PIP.

**Results:**

Four hundred and twenty eight from 531 patients with known therapy for cardiovascular diseases (CVDs) were included in the analysis of PIP (40.52% aged 65–69 years, 61.88% female, 64% had up to 6 comorbidities, and 21.72% presenting with polypharmacy). A total of 71 PIMs in 64 patients with polypharmacy were identified during applying STOPP criteria. 56% of patients taking above five medicines daily had PIMs. The majority of PIMs (31%) were related to CVDs treatment, followed by PIMs in the treatment of endocrine diseases (22.54%), duplication of medicines (8.46%) and prolonged treatment with benzodiazepines (8.46%). Forty four PPOs were identified with START criteria. 22.72% were related to lack of proton pump inhibitors (PPI) in the presence of gastroesophageal disorders, and the same percentage was for lack of Calcium-vitamin D supplementation in osteoporosis. Applying the methodology of risks calculation the sample risk for PPO was 2.1% and for PIM 3.4%. At sample level the relative risk for PPO was 62% out of the risk for PIM and at population level varied between 42.8 and 89.8% and it is statistically significant. The number needed to treat for the event to happen is 77.5, meaning that at every 78 prescriptions there is a chance to appear PIP.

**Conclusion:**

Application of methodologies for detection of potentially inappropriate prescribing is not part of routine clinical practice in Bulgaria. Our study demonstrates a high percentage of potentially inappropriate medication among older patients with polypharmacy. Along with the aging population in Bulgaria, economic burden of polypharmacy and the prevalence of cardiovascular diseases, it is especially important to address potentially inappropriate medication use in cardiovascular patients. There is a considerable necessity for implementation of measures for early detection of potentially inappropriate medication and potentially prescribing omission as a part of de-prescribing strategies in older patients.

## Introduction

Polypharmacy is considered as the concurrent use of multiple medications. There is no standard definition, but the most commonly accepted among authors is the routine use of five or more medications. The over-the-counter, prescription and/or traditional and complementary medicines used by a patient are also counted in the definition (1). Older people are at increased risk of polypharmacy due to the prevalence of chronic diseases, multiple prescribers, and simultaneous use of drugs, which can compromise their health performance, can lead to adverse drug reactions, drug interactions, non-adherence to therapy, hospitalizations, systemic disorders and increased mortality (2–4). When polypharmacy is unavoidable the key challenges are to achieve evidence-based appropriate use of medicines, especially in older people with multimorbidity.

Cardiovascular diseases (CVDs) are the leading cause of death worldwide, with an estimated 17.9 million lives each year. In Europe in 2017, above 20% of the population was aged ≥60 years, and the expected increase is to 35% by 2050. The incidence of CVDs increases with age. From 40% in adults aged 40–59 years, to 75% in those 60–79 years, and 86% in those >80 years, experienced CVDs (5). According to statistical data, Bulgaria ranks first in Europe and third in the world in mortality from cardiovascular diseases (6).

Older patients have a higher prevalence of CVDs, high rates of CV risk factors, and multiple age-related comorbidities. Drug prescribing in the older is complicated by comorbidity, polypharmacy and age-specific changes in pharmacokinetics and pharmacodynamics. All of these factors make managing efficacy and safety in older patients with CVDs a challenging and complex process, which poses an increased risk of potentially inappropriate prescribing (PIP) (7). PIP refers to prescribing medications that may not produce benefits relative to harm, or not prescribing medications that are recommended. PIP includes Potentially inappropriate medication (PIMs) and potential prescribing omissions (PPOs). PIMs are medications with a greater risk than benefit to a patient, while PPOs are failures to prescribe medications of potential benefit (8, 9).

In order to evaluate and avoid PIPs several methodologies have been developed to help physicians and health care professionals have the possibility to quickly check PIMs, as well as to detect PPOs. One of the well-known and used tool in Europe for detection of both PIMs and PPOs is the explicit STOPP (Screening Tool of Older Person's Prescriptions)/START (Screening Tool to Alert doctors to Right Treatment). STOPP/START criteria were first published in 2008 and later in 2015 were updated. They are designed as “evidence-based explicit criteria for common and/or important instances of PIMs (STOPP) and PPOs (START), arranged according to physiological systems as in drug formularies” (10).

Although the use of multiple drugs is part of recommended strategy for treatment of CVDs, it may increase the risk for PIMs (11).

The safety of prescribing in older patients is the subject of serious research regarding appropriate prescriptions, combinations, and drug-related issues.

In Bulgaria, drug related problems are not evaluated, namely the patterns of prescription and use of medicines in adults population over 65 years of age. Considering that CVDs are the leading cause of death among older patients, we intended to determine the prevalence of PIMs and PPOs, defined by the STOPP/START criteria, and to investigate the relationship between the number of medications taken and the possibility of occurrence of PIPs.

## Materials and methods

### Study design and patient recruitment

A national representative, prospective questionnaire study among patients over 65 years of age was conducted in 25 pharmacies in Bulgaria.

Pharmacies were randomly selected, depending on the pharmacy manager's willingness to participate, throughout the whole country. Graduating pharmacy students conducted the interviews with patients after informed consent was obtained. The sample size of 384 people was considered as satisfying the 5% confidence level for a population 1.5 million older Bulgarian inhabitants in the country. Representativeness was ensured by selecting patients that agreed to participate and belongs to the selected geographical areas. Three main geographical centers were chosen as coordinating centers – Varna (*n* = 7 pharmacies), Sofia (*n* = 11 pharmacies), and Plovdiv (*n* = 7 pharmacies), corresponding to the eastern, middle and western parts of the country, with a total population of 3 million (50% of the population). Each center selected pharmacies from the main city and other settlements with the highest workload, in order to ensure sufficient recruitment options and representativeness. Pharmacies in Sofia recruited 29 patients per pharmacy, 19 in Varna and 12 in Plovdiv, respectively. Questioners were instructed to collect information for 1 patient per day for a period of 1 month at least.

The questionnaire was developed for the EUROAGEISM project, funded under the Horizon 2020 program (12). The questionnaire is rather detailed and included data on demographic characteristics, lifestyle, motor activity, laboratory tests performed by patients, patient health status, use of health services, existing diseases, symptoms of disease manifestation, pain and depression, medications taken and satisfaction with therapy.

Five hundred and forty three patients were recruited, and 531 had available and relevant prescription information extracted from the questionnaire. Evaluation of PIMs and PPOs was performed for 428 patients who have a CVDs prescription. Based on the information in the questionnaire all STOPP/START criteria were applied.

According to World health organization the “Global Atlas on Cardiovascular Disease Prevention and Control” cardiovascular disease (CVD) is a class of diseases that involve the heart or blood vessels (13) and include coronary artery diseases (CAD) such as angina and myocardial infarction, stroke, heart failure, hypertensive heart disease, rheumatic heart disease, cardiomyopathy, abnormal heart rhythms, congenital heart disease, valvular heart disease, carditis, aortic aneurysms, peripheral artery disease, thromboembolic disease, and venous thrombosis. In this study we analyzed only medicines prescribed for patients with cardiovascular diseases, presented on the second part of **Table 2** because only those diagnoses were reported.

### Inclusion and exclusion criteria

The survey included patients above 65 years old who received their medicines from the relevant pharmacy and agreed to participate. The age limit of 65 years was stated at the EuroAgeism project and corresponds with the definition of OECD that the older population are people aged 65 and over (14). The inclusion criteria were the age of patients, willingness to participate and answer the questions. No limitations were defined.

### Ethics

Every patient was acquainted with the purposes of the study, and their informed consent was obtained. The study was approved by the ethics committees of the Medical Universities in Sofia (737/20.02.2019), Plovdiv (04.02.2019) and Varna (161/07.02.2019). The ethical approvals are available upon request from the authors.

### Evaluation of PIPs, PPOs, and PIMs

PIP (potentially inappropriate prescribing) was evaluated as a drug related problem that encompasses potentially inappropriate medication (PIM) and Potential prescribing omissions (PPO) (15, 16).


$$\begin{array}{l} PIP=PIM+PPO \end{array}$$

(17, 18).

The STOPP/START criteria version 2015 was used for the evaluation of PIMs and PPOs and then the resulting PIPs were calculated (15–20). All prescriptions for the CVDs under consideration were evaluated by two members of the research team for their correspondence with the definitions set by STOP/START system.

### Risk for PIP, PPOs, PIMs calculation

The two events—PIMs and PIPs were evaluated for their risks, based on the recommendations illustrated by Sacket, Richardson, Rosenberg and Haynes in Evidence based medicine (21).

The main questions explored were which event was more likely to occur, a PIM or a PPO and what is the relationship between the risks and odds, using the following formulae.


$$\begin{array}{l} Absolute\;risk\;reduction\;\left(ARR\right)=p2-p1\left(\%\right)\\ \;Relative\;risk\;\left(RR\right)=p1/p2\\ \;Risk\;reduction=1-RR\left(\%\right)\\ Odds\;ratio\;\left(OR\right)=r2\left(n2-r2\right)/r2\left(n1-r1\right). \end{array}$$

Where p2 and p1 are the proportions (%) of patients with a PIM or PPO, respectively. Number of patients in groups is n1 and n2, respectively and r1, r2 is the number of patients with event (PPO, or PIM).

## Results

### General characteristics

The demographic characteristics of patients are shown in Table 1. Majority of respondents were female (61.88%), with the highest proportion being patients between 65 and 69 years (40.52%). Polymorbidity is present in our observed sample as well. A total of 3,130 diseases or symptoms were reported, an average of 6 per respondent, with 64 percent having up to 6 symptoms or diseases. It should be noted that some symptoms were related to an already reported disease. There was no patient who was disease free, which can be contributed to the fact that these patients were visiting the pharmacy to collect a valid prescription.

**Table 1:** Demographic characteristic of study population.

**Characteristics of patients (*N* = 543)**	
**Gender**	***n*** **(%)**
Male	189 (34.8)
Female	336 (61.88)
No information for medication therapy	18 (3.32)
**Age groups**	***n*** **(%)**
65–69 years	220 (40.52)
70–74 years	145 (26.7)
75–79 years	77 (14.18)
80–84 years	54 (9.95)
above 85 years	40 (7.37)
No data	7 (1.28)
**Reported diseases and symptoms**	***n* (%)**
1–3	178 (33)
4–6	166 (31)
7–10	134 (28)
11–15	63 (8)

The most reported diseases and symptoms among respondents were; diseases of the cardiovascular system (*n* = 816), sensory organs (hearing and sight, *n* = 452) and mental diseases (*n* = 438). Hypertension was the leading diagnosis among cardiovascular diseases, with more than half of patients (*n* = 383) reporting being diagnosed (Table 2). Some patients reported multiple CVD diseases, which is accounted by the higher number of present CVDs than actual patients—due to multimorbidity.

**Table 2:** Reported diseases.

**Reported diseases**	***N***
CVDs	816
Musculoskeletal	306
Endocrine and metabolic disorders	326
Respiratory disorders	60
Digestive system	317
Genitourinary	169
Blood disorders	44
Neurological	40
Psychiatric disorders	438
Infectious diseases	64
Cancer diseases	19
Vision and hearing	452
Skin diseases	29
Other diseases	50
**CVD diseases**	
Hypertension	383
Angina ischemic heart disease	97
Myocardial infarction	43
Heart failure	89
Stroke	44
AV blockage	7
Arrhythmia	97
Deep vein thrombosis	28
Pulmonary embolism	5
Ischemic leg disease	7
Bleeding history	16

### Medication profile and identification of PIPs

On total, 2092 medications were reported by patients, including food supplements, with an average of 3.98 per patient. 64.76% took between 2 and 5 medications per day, while the percentage of patients with polypharmacy was 21.72%, with only 1.91% taking more than ten medications daily (Table 3).

**Table 3:** Prescribed type and number of medication therapy.

**Type of medication**	***N***
Medicinal products	2,004
Food supplements	88
Total	2,092
Mean	3.98
**Number of medications**	***N*** **of patients (%)**
1	71 (13.52)
2–5	340 (64.76) out of them 33% with five medicines
6–10	104 (19.81)
>10	10 (1.91)

A total of 71 PIMs in 64 patients with polypharmacy were identified when applying STOPP criteria. 56% of patients taking above five medicines daily had PIMs. 22 PIMs were related to prescriptions for CVD treatment; in terms of endocrine system diseases, there were 16 PIMs, followed by duplication of medicines (*n* = 6) and more prolonged treatment with benzodiazepines (*n* = 6) (Table 4).

**Table 4:** Screening tool of older people's potentially inappropriate prescriptions (STOPP).

**Indication of medication**	***N***
Any duplicate drug class prescription e.g., two concurrent NSAIDs, SSRIs, loop diuretics, ACE inhibitors, anticoagulants (optimization of monotherapy within a single drug class should be observed prior to considering a new agent)	6
**Cardiovascular system**	
Digoxin for heart failure with normal systolic ventricular function (no clear evidence of benefit)	6
Loop diuretic as first-line treatment for hypertension (safer, more effective alternatives available)	4
Loop diuretic for treatment of hypertension with concurrent urinary incontinence (may exacerbate incontinence)	2
Centrally-acting antihypertensives (e.g., methyldopa, clonidine, moxonidine, rilmenidine, guanfacine), unless clear intolerance of, or lack of efficacy with, other classes of 2 antihypertensives (centrally-active antihypertensives are generally less well-tolerated by older people than younger people)	2
Aldosterone antagonists (e.g., spironolactone, eplerenone) with concurrent potassium conserving drugs (e.g., ACEI's, ARB's, amiloride, triamterene) without monitoring of serum potassium (risk of dangerous hyperkalaemia i.e., > 6.0 mmol/l – serum K should be monitored regularly, i.e., at least every 6 months)	8
**Antiplatelet/anticoagulant drugs**	
Aspirin with a past history of peptic ulcer disease without concomitant PPI (risk of recurrent peptic ulcer)	3
NSAID with concurrent antiplatelet agent(s) without PPI prophylaxis (increased risk of peptic ulcer disease)	2
**Central nervous system and psychotropic drugs**	
Benzodiazepines for ≥4 weeks (no indication for longer treatment; risk of prolonged sedation, confusion, impaired balance, falls, road traffic accidents; all benzodiazepines should be withdrawn gradually if taken for more than 4 weeks as there is a risk of causing a benzodiazepine withdrawal syndrome if stopped abruptly)	6
**Renal System (The following drugs are potentially inappropriate in older people with acute or chronic kidney disease with renal function below particular**	
**levels of eGFR)**	
Digoxin at a long-term dose >125 μg/day if eGFR <30 ml/min/1.73 m2 (risk of digoxin toxicity if plasma levels not measured)	1
**Gastrointestinal system**	
Drugs likely to cause constipation (e.g., antimuscarinic/anticholinergic drugs, oral iron, opioids, verapamil, aluminum antacids) in patients with chronic constipation where non-constipating alternatives are available (risk of exacerbation of constipation)	2
Oral elemental iron doses >200 mg daily (e.g., ferrous fumarate> 600 mg/day, ferrous sulfate > 600 mg/day, ferrous gluconate> 1,800 mg/day; no evidence of enhanced iron absorption above these doses)	1
**Respiratory system**	
Theophylline as monotherapy for COPD (safer, more effective alternative; risk of adverse effects due to narrow therapeutic index)	1
**Musculoskeletal system**	
NSAID with severe hypertension (risk of exacerbation of hypertension) or severe heart failure (risk of exacerbation of heart failure)	1
Long-term use of NSAID (>3 months) for symptom relief of osteoarthritis pain where paracetamol has not been tried (simple analgesics preferable and usually as effective for pain relief).	2
Long-term NSAID or colchicine (>3 months) for chronic treatment of gout where there is no contraindication to a xanthine-oxidase inhibitor (e.g., allopurinol, febuxostat) (xanthineoxidase inhibitors are first choice prophylactic drugs in gout)	1
COX-2 selective NSAIDs with concurrent cardiovascular disease (increased risk of myocardial infarction and stroke)	2
Oral bisphosphonates in patients with a current or recent history of upper gastrointestinal disease i.e., dysphagia, oesophagitis, gastritis, duodenitis, or peptic ulcer disease, or upper gastrointestinal bleeding (risk of relapse/exacerbation of oesophagitis, esophageal ulcer, esophageal stricture)	1
**Endocrine system**	
Sulphonylureas with a long duration of action (e.g., glibenclamide, chlorpropamide, glimepiride) with type 2 diabetes mellitus (risk of prolonged hypoglycaemia)	8
Beta-blockers in diabetes mellitus with frequent hypoglycaemic episodes (risk of suppressing hypoglycaemic symptoms)	8
**Drugs that predictably increase the risk of falls in older people**	
Hypnotic Z-drugs e.g., zopiclone, zolpidem, zaleplon (may cause protracted daytime sedation, ataxia)	3
**Analgesic drugs**	
Use of regular (as distinct from PRN) opioids without concomitant laxative (risk of severe constipation)	1
Total PIM	71

Forty four PPOs in 44 patients were detected applying START criteria. The majority of PPOs were detected in the gastrointestinal system—lack of PPI in the presence of GERD (*n* = 10) and lack of fiber supplements when a patient had a history of constipation (*n* = 10). 15 PPOs were related to treatment omissions regarding the muscle-skeletal system, with the majority related to skipping Vitamin D as a supplement when the patient is with osteoporosis/osteopenia (*n* = 10) (Table 5).

**Table 5:** Screening tool to alert doctors to right i.e., appropriate, indicated treatment (START).

**Cardiovascular system**	**Nr:**
Antiplatelet therapy (aspirin or clopidogrel or prasugrel or ticagrelor) with a documented history of coronary, cerebral or peripheral vascular disease	1
Angiotensin Converting Enzyme (ACE) inhibitor with systolic heart failure and/or documented coronary artery disease	1
Beta-blocker with ischemic heart disease	2
Appropriate beta-blocker (bisoprolol, nebivolol, metoprolol or carvedilol) with stable systolic heart failure.	1
**Respiratory system**	
Regular inhaled b2 agonist or antimuscarinic bronchodilator (e.g., ipratropium, tiotropium) for mild to moderate asthma or COPD	1
**Gastrointestinal system**	
Proton Pump Inhibitor with severe gastro-esophageal reflux disease or peptic stricture requiring dilatation	10
Fiber supplements (e.g., bran, ispaghula, methylcellulose, sterculia) for diverticulosis with a history of constipation	10
**Musculoskeletal system**	
Vitamin D and calcium supplement in patients with known osteoporosis and/or previous fragility fracture(s) and/or (Bone Mineral Density T-scores more than−2.5 in multiple sites)	7
Vitamin D supplement in older people who are housebound or experiencing falls or with osteopenia (Bone Mineral Density T-score is > −1.0 but <−2.5 in multiple sites)	3
Xanthine-oxidase inhibitors (e.g., allopurinol, febuxostat) with a history of recurrent episodes of gout	3
Folic acid supplement in patients taking methotrexate	2
**Endocrine system**	
ACE inhibitor or Angiotensin Receptor Blocker (if intolerant of ACE inhibitor) in diabetes with evidence of renal disease i.e., dipstick proteinuria or microalbuminuria (>30 mg/24 h) with or without serum biochemical renal impairment	2
**Analgesics**	
Laxatives in patients receiving opioids regularly	1
Total PPO	44

PPOs were not identified regarding central nervous system and eyes, urogenital system and vaccines. In terms of vaccines, it is worth emphasizing that we cannot accurately detect these criteria since immunization with influenza and pneumococcal vaccines is not mandatory for older adults.

The potentially inappropriate prescribing was detected for 115 (71PIMs +44 PPOs) prescriptions. These numbers were included in the calculations of the risk for their appearance in prescriptions (Tables 6, 7).

**Table 6:** Risks for development of PIMs, and PPOs for the observed prescriptions.

**Group 1**	**Group 2**
Number of prescriptions with event PPO r1 (44)	Number of prescriptions with event PIM r2 (71)
Number of prescriptions in the group n1 (2092)	Number of prescriptions in the group n2 (2092)
Proportion (%) of prescriptions with event in group 1 PPO, (p1)	2.1
Proportion (%) of prescriptions with event in group 2, PIM (p2)	3.4
Absolute risk reduction (ARR)	1.3
Standard error (SE) of ARR	0.5
95% confidence interval (CI) for ARR	0.301–2.281
Number needed to treat (NNT)	77.5
95% confidence interval (CI) for NNT	43.85–332.68
Relative risk (RR)	0.62
95% confidence interval (CI) for RR	0.43–0.898
Relative risk reduction (RRR)	38
95% confidence interval (CI) for RRR	10.2–57.20
Odds ratio (OR)	0.612
95% confidence interval (CI) for OR	0.418–0.895

**Table 7:** Risks for PIMs, and PPOs at patient level.

**Group 1**	**Group 2**
Number of patients with event PPO r1 (44)	Number of patients with event PIM r2 (71)
Number of patients in the group n1 (428)	Number of patients in the group n2 (428)
Proportion (%) of patients with event in group 1 PPO, (p1)	10.29
Proportion (%) of patients with event in group 2, PIM (p2)	16.6
Absolute risk reduction (ARR)	6.3
Standard error (SE) of ARR	2.3
95% confidence interval (CI) for ARR	1.759–10.858
Number needed to tear (NNT)	15.852
95% confidence interval (CI) for NNT	9.21–56.855
Relative risk (RR)	0.62
95% confidence interval (CI) for RR	0.436–0.881
Relative risk reduction (RRR)	38
95% confidence interval (CI) for RRR	11.89–56.399
Odds ratio (OR)	0.576
95% confidence interval (CI) for OR	0.385–0.862

Most PIMs (*n* = 22) were related to CVDs treatment. The most common detected PIMs in this class were related to prescription of Digoxin for heart failure, Furosemide, Torasemide as first line treatment for hypertension and the combination of Spironolactone with ACEIs and ARBs.

In terms of endocrine system there were 8 detected inappropriate prescriptions associated with glibenclamide or glimepiride in patients with diabetes type 2, as common comorbidity to hypertension. Eight PIMs are linked to beta blockers (metoprolol, bisoprolol). There were 6 patients who had duplication of drug class medications (simultaneous use of 2 NSAIDS - diclofenac, ibuprofen for pain relief). The prolonged use of bromazepam, alprazolam was also observed (*n* = 6).

The sample risk for PPO is 2.1%, and for PIM is 3.4%. The absolute risk reduction /ARR=p2-p1/ for PIM and PPO at sample level is 1.3% which means that patients have a 1.3% higher risk of being prescribed an inappropriate medication as opposed to having a prescription omission. Confidence intervals put the population risk to be between 0.301 and 2.281%. The relative risk between groups in our sample was calculated to be (for PPO) 62% out of the risk for PIMs, with the confidence interval putting the population risk between 42.8 and 89.8%, which was statistically significant. Based on these numbers, the combined risk of a PIP (PIM+PPO) will be 5.5%, varying between 4.562 and 6.565% at the population level.

The number needed to treat for the event to happen is 77.5, translating to a PIP once every 78 prescriptions.

Curiously, all 71 PIMs and 44 PPOs were unique, meaning that 115 individual patients experienced PIP which allows for similar risk calculations to be conducted at the patient level (*n* = 428), which allows us the risk of assessing the odds and risks for a PIP on an individual level, as opposed to per prescription. Importantly, what these calculations show is that a PIP could occur once every 15.8 patients (Table 7).

The PIP as a sum of the PIM and PPO proportions is 26.89% (10.29 + 16.6%). If we relate those results to the general country population of 1.73 million above the age of 65 years, we might expect that 457,130 adult will have PIP.

## Discussion

This is the first study in Bulgaria assessing the potential inappropriate prescribing in older patients with CVDs. The assessment tools for PIPs are not used in Bulgarian practice, although various studies in Europe and worldwide prove their usefulness in clinical practice (11).

Recently the terms “appropriate polypharmacy” and “inappropriate polypharmacy” have gained a widespread use. 2019 WHO Medication Safety Technical report provides some clarification on both terms (22). Having in mind that the most commonly accepted among authors definition that polypharmacy is the routine use of five or more medications, while 10 or medications is defined as excessive polypharmacy, we used these values as cutoff points. However, in our sample we also observe patients taking various medications. 22% of the recruited patients receive more than 6 medicines and 65% take between 2 to 5, but out of them 33% take 5 medicines, which allows for the possibility of inappropriate prescribing within this population as well. This requires paying a special attention to older patients with polypharmacy and implementing a measure for tracking and evaluation of PIPs among older patients. We also have to clarify that 88 people take OTC and dietary supplements that are included in the definition.

A study among 28 countries using data from the WHO showed that multimorbidity is a global phenomenon that already affects middle- and low-income countries, with an estimated average prevalence of 7.8% for these countries, and among higher income countries, this percentage varies between 8.3 and 27% (23). An Australian study of 4,574 patients found that comorbid and polymorbid conditions were more common among the older over 60 years of age, with an average of 2.4 co-morbid conditions being the most frequent (24). These results are also confirmed in Canada (25), where in people over the age of 25, the frequency of comorbidity is only 10%, while in older patients over 65 this percentage rises to 62%, and in the USA, where the authors found that 65% of adult patients have more than 2 diseases (26). Similar are the results in our study where polymorbidity affects almost all of people above 65 years of age.

As was pointed above the OTC and dietary supplements intake have to be considered as part of the whole medication. An analysis of OTC drug utilization among the older population found that in 2011, about 67% took 5 or more drugs in combination with their standard treatment (27), with self-medication by these patients placing about 15.1% at risk of serious drug interactions, caused by a contraindication between the medicinal product and non-prescription drugs or nutritional supplements, and again, this trend is in a positive direction and continues to strengthen (28). The risk of the patient not reporting the supplements and OTC products they are taking is a complicating factor. Jou and colleagues estimated the percentage of patients who did not share with their treating physicians about their self-medication at 25% (29). In our study the reported use of supplements is relatively low. Because we have a very few data for OTC and dietary supplements intake, which might be underreported by patients, it stands to reason that the actual number of polypharmacy patients could be higher.

Latvian researchers showed that prevalence of PIM varies from 24 to 26 to 57% (30) depending on the version of Beers criteria used: 2003 or 2015, respectively (31, 32). A retrospective study among 780 older patients using STOPP/START criteria in Taiwan showed that 39% from the patients had at least one PIM. Multivariate analysis revealed that PIM risk was associated with the number of medications prescribed (*P* < 0.001) and the presence of cardiovascular (*P* < 0.001) or gastrointestinal disease (*P* = 0.003) (33). We found that the PIPs in the observed Bulgarian population are similar than that in Latvia. Unfortunately, in Bulgaria there is no system on place for their evaluation like STOPP/START criteria.

Previous study performed a meta-analysis of the reported risks for PIPs and related risk for Bulgaria (34). It calculated that 244,227 people are exposed to risk for PIP in the population above 65 years of age. Our study shows that the real risk is with 167,697 people more than calculated based on literature data. These results must raise concern for health care professionals, both pharmacy and physicians.

A recent study in Serbia applied Beers criteria for detecting PIMs among 1,500 older patients with CVDs. The results showed that the PIM frequency in the older population was 70.3%, more frequent in female elders. Several risk factors were pointed out as polypharmacy, gender, nicotine use, cognitive status, nutrition state, as well as the number of diseases in the study sample (35). Ubeda et al. conducted a review of the medication and clinical records of 81 residents in nursing home in Spain, using Beers and STOPP/START criteria, v01. The Beers criteria identified potentially inappropriate medication use in 25% of patients and 48% of patients used at least 1 inappropriate medication according to STOPP criteria. START detected 58 potential prescribing omissions in 44% of patients. Calcium-vitamin D supplementation in osteoporosis was the most frequent rule (15%), but omissions corresponding to the cardiovascular system implied 23% of patients (36). Our research also confirmed that one of the most common PPO is exactly associated with Calcium Vitamin D supplements. Our study is in accordance with those results.

A cross-sectional retrospective study (PIM-CCVAE) was performed at a secondary healthcare level in Brazil to identify the use of PIMs focusing on cardiovascular and cerebrovascular Adverse Events. The study showed that 74.2% of older patients used at least one PIM-CCVAE and were taking daily 1.3 (±1) PIM per older. The most significant factors associated with intake of PIM-CCVAE were found to be the presence of comorbidities, cardiovascular diseases, polypharmacy, and low to moderate morbidity and mortality scores (37).

Several studies, focused on the detection of PIMs, identified PPIs above maintenance dosage for >8 weeks as most frequent PIM (38–40). It's worth mentioning that our study did not support these results most likely because our patients are primarily with CVD. Hence, further studies need to be done to identify if this is a common PIM in Bulgaria as well.

Analysis of the European project EUROAGEISM H2020, FIP7 program was performed aiming evaluation of approval rates and marketing of EU (7)-PIM criteria compared to AGS Beers 2015 criteria in six EU countries. The research showed that the lack of evidence on PIM prescribing in older patients in different level of settings of healthcare, especially in Central and Eastern Europe, contributes to probably still higher rates of inappropriate prescribing of PIMs in many countries. High specificity of these criteria was determined for the pharmaceutical market of a country that contributed to the development of the EU (7)-PIM list (ES). Authors also pointed out that the criteria are with a low specificity in Eastern and Central EU countries, and that more research effort should be devoted in this area (41).

Applying STOPP/START criteria is admittedly valuable for the practice of HCPs and pharmacists; however, when it is paper-based, it is time-consuming. After developing STOPP/START criteria, more effort was put into the electronic deployment, which is highly desirable for use in routine practice. Two clinical trials (SENATOR and OPERAM) involve the fully electronic deployment of STOPP/START criteria. They used diagnostic and medication coding systems, supplemented by other patient data that quantify functional and cognitive status and laboratory test results. However, considering that currently, the number of criteria in version 2 is 114, experts supposed that there is a need to train health professional to be able critically to interpret the results of the STOPP/START criteria, related to any particular case (22).

Although it is beyond the scope of the paper to analyze the reasons behind PIPs, several possible causes for inappropriate prescribing can be speculated. As previously stated, there is a lack of any tool for evaluation of the prescriptions. Reimbursement authorities introduced electronic prescriptions just some months ago, without ways or policies to estimate incorrect prescribing. Furthermore, older patients often visit diverse specialists, and the country does not have mechanisms to control rational prescribing, neither does it offer supplementary education regarding rational drug use or prescribing. This is true on all levels of care – from physicians, to students, to patients. We hope that with this study, we can highlight the dangers this poses and draw the attention of physicians and authorities to the importance of the PIPs and risks for the society, in order to, hopefully, enact measures to improve the situation. According to the report of the National Statistics Institute in Bulgaria the EU's population is aging. The data show an increase in the share of the older (over 65). From 16% in 2001, they reached 21% in 2020—an increase of 5 percentage points. During the period 2001–2020, there was an increase in the proportion of persons aged 65 and over in all Member States (42). The study population included in the current analysis show that all patients have a CVDs and the majority of PIMs are related to treatment of these diseases, especially in older with polypharmacy. It is essential for all health care professionals to increase awareness and understanding of potential PIM use by cardiovascular patients. Based on that we can expect that the risk will increase in the future.

Limitations of our study are that it was performed only with pharmacy visitors in a relatively independent health condition. We do not include people in nursing homes or hospitals where we can expect higher polypharmacy and multimorbidity. The BEERs and STOPP/START criteria are widely used but not customized for Bulgaria that also might be considered as limitation to the study.

## Conclusion

Polypharmacy and associated PIPs are common among older patients. In the past 10 years experts focus on drug related problems and the possibilities for avoiding them. Several tools for identification of potential inappropriate medications have been developed aiming to encourage the geriatrics and other HCPs to review in more detail the prescriptions in older population. Along with the aging population in Bulgaria, economic burden of polypharmacy and the prevalence of CVDs it is especially important address PIMs use in cardiovascular patients. There is a considerable necessity for implementation of measures for early detection of PIMs and PPOs as a part of de-prescribing strategies in older patients.

## Data availability statement

The raw data supporting the conclusions of this article will be made available by the authors, without undue reservation.

## Ethics statement

The studies involving human participants were reviewed and approved by Ethical Commitees at the MUS, MUP, MUV. The patients/participants provided their written informed consent to participate in this study.

## Author contributions

TK collected data, calculated variables, and analyze and wrote the first drafts. PM calculated PIPs, validated data, and wrote first draft and final text. KT design the study, calculate risks, analyze information, and wrote final version. KM performed statistical analysis, interpret statistics, and validate data. GP design the study, create methodology, analyze data, and wrote final text. All authors contributed to the article and approved the submitted version.
